# Palladium Supported on Calcium Decorated Carbon Nanotube Hybrids for Chemoselective Hydrogenation of Cinnamaldehyde

**DOI:** 10.3389/fchem.2019.00751

**Published:** 2019-11-13

**Authors:** Ying Ma, Lu Feng, Zhanglong Guo, Jiangtao Deng, Cuong Pham-Huu, Yuefeng Liu

**Affiliations:** ^1^Dalian National Laboratory for Clean Energy (DNL), Dalian Institute of Chemical Physics, Chinese Academy of Sciences, Dalian, China; ^2^University of Chinese Academy of Sciences, Beijing, China; ^3^Zhang Dayu School of Chemistry, Dalian University of Technology, Dalian, China; ^4^Institute of Chemistry and Processes for Energy, Environment and Health (ICPEES), UMR 7515 CNRS-University of Strasbourg, Strasbourg, France

**Keywords:** Pd nanoparticles, Ca promoter, electronic modification, cinnamaldehyde hydrogenation, monolithic nanocarbon materials

## Abstract

The chemoselective hydrogenation of cinnamaldehyde (CAL) to the corresponding hydrocinamaldehyde (HCAL) is a type of important reactions in fine chemistry, which is critically dependent on the rational design the chemical structure of active metal. In this work, calcium promoted palladium on CNT hybrid (Ca-Pd@CNT) with monolithic structure was synthesized through one-pot alginate gel process. The catalytic performance results showed that moderate Ca promotion catalyst (Ca-Pd@CNT_HCl−2h_) present a superior CAL hydrogenation activity with CAL conversion of 99.9% and HCAL selectivity of 86.4% even at the lager Pd nanoparticle size (c.a. 5 nm). The characterization results show that the electron transfer between the additive Ca promoter and Pd nanoparticles (NPs) could modify the electron structure of Pd species and induce the formation of the partial positively charged Pd^δ+^ species on the Pd NPs surface in the Ca-Pd@CNT_HCl−2h_ catalyst resulting to the satisfactory catalytic performance. Furthermore, the one-pot gel synthesis methodology for microscopic carbon supported catalyst could also endows its great potential industry application in heterogeneous catalysis with easily handling during the transportation and reaction, and attributed to reducing the overall pressure drop across in the fix-bed reactor.

## Introduction

Catalytically selective hydrogenation of α, β-unsaturated aldehydes via C=O or C = C bond to α, β-unsaturated alcohols or saturated aldehydes is considered as an important fine chemical process for producing intermediates for food additives and pharmaceuticals (Gallezot and Richard, 1998; Mäki-Arvela et al., 2005; Wu et al., 2012; Daly et al., 2014; Kahsar et al., 2014; Tian et al., 2015). However, the C = C and C=O bond are conjugated in the typical substrate of α, β-unsaturated aldehydes-cinnamaldehyde (CAL), which result in a mixture of hydrogenation products such as cinnamyl alcohol (COL), hydrocinnamaldehyde (HCAL), and 3-phenyl propanol (PPL). Palladium-based catalyst is favor to C = C hydrogenation due to its special position of d-band center. Therefore, Pd could work as the active metal center for hydrogenation of CAL to produce the HCAL (Garcia-Mota et al., 2010; Ide et al., 2012; Cárdenas-Lizana et al., 2013; Arrigo et al., 2014; Salnikov et al., 2014; Durndell et al., 2015; Zhao et al., 2015). Tailoring the electronic structure of Pd active sites through adjustment surface chemistry of catalyst support (Balmes et al., 2012; Cárdenas-Lizana et al., 2013; Figueiredo, 2013; Arrigo et al., 2016; Rao et al., 2017), construction of the bimetallic or alloy composition (Vu et al., 2006; Tsang et al., 2008), regulation of the particle size (Amorim and Keane, 2008; Jiang et al., 2016; Xu et al., 2019) and the addiction of promoter (Tsang et al., 2008) could promote the CAL hydrogenation activity as well as the HCAL selectivity (Toebes, 2004; Jiang et al., 2016). The alkalis and alkaline-earth metals, such as Li, Na, K, Rb, and Sr, are reported as effective promoter cations for CAL hydrogenation (Galvagno et al., 1986; Li et al., 1997; Hsu et al., 2010; Bhogeswararao and Srinivas, 2012), which could increase the HCAL selectivity. Furthermore, alkalis and alkaline-earth metals as promoter could also modify the coordination environment around the noble metal atom through charge transfer between the active metal and the promoter, even forming new catalyst sites and thus regulating the catalytic properties (Song et al., 2006). Yang et al. (2017) found that the controlled addition of potassium with Pt/zeolite catalysts can create the Pt-O (OH)-K interfacial sites, which could greatly accelerated the reverse water gas shift reaction. Wei et al. (2017) also prepared Na cations modified the Pt/FeO_x_ catalyst and the formed Pt-O-Na-O-Fe-like species could enhance the 3-aminostyrene selectivity significantly while the hydrogenation activity of 3-nitrostyrene remained unchanged. Considering the alkalis metal can act as the electronic modifiers, we proposed to regulate the activity and selectivity of cinnamaldehyde hydrogenation by controllable introducing Ca to modify the Pd-based catalyst and investigate the influence of calcium promoter. Meanwhile, the reaction rate could also be reduced due to the blocking or poisoning the active sites with inappropriate loading amount of Ca (Yang et al., 2017).

Compared to conventional metal oxides, carbon is an inert support, which can be used as a platform to disperse and study the changes of electronic state of metals (Auer et al., 1998; Su et al., 2013). Carbon nanotubes (CNT) are widely used in heterogeneous catalysis due to their chemical stability, low cost, and easily recovery of precious metals by means of combustion and other special properties in recent years C (Arrigo et al., 2012; Gao et al., 2018; Zhu et al., 2019). However, it is difficult to separate CNT supported catalysts after the liquid reaction due to its original powder form. Therefore, a macroscopic shaped CNT-based catalyst would facilitate the catalyst separating and handling which present a great potential either in the liquid phase and gas phase heterogeneous catalytic reactions (Liu et al., 2013, 2017; Ba et al., 2017; García-Bordejé et al., 2017).

In this work, monolithic CNT supported Pd catalyst is synthesized by one-pot process via combined palladium nitrate, CNT and alginate solution. During the synthesis process, the alginate played as biopolymer template to form alginate gel in the presence of CaCl_2_ solution with a homogeneous size and macroscopic shape. The obtained monolithic Ca-Pd@CNT beads with different Ca promoter concentration were employed as catalyst for the hydrogenation of CAL to HCAL. The monolithic structural carbon-based catalysts could be easily separated from the liquid reactant and thus facilitates the catalysts recycling. The structural and electronic properties of such monolithic Ca-Pd@CNT catalyst are analyzed by different characterization techniques to understand the structure-activity relationship in the presence of Ca promoter.

## Experimental Section

### Materials

The commercial multi-walled carbon nanotubes (CNT) were pre-treated by hydrochloric acid (6 mol/L) at room temperature for 12 h in order to remove residual metal impurities. CaCl_2_, alginate and hydrochloric acid were purchased from Tianjin Kemiou Chemical Reagent Co., Ltd. (Tianjin, China). Pd (NO_3_)_2_·2H_2_O was purchased from Alfa Aesar. All chemical agents were used as received without further purification.

### Catalyst Preparation

Macroscopic shaped CNT beads (Liu et al., 2012; Ba et al., 2016):1 g of CNT was dispersed in 50 mL distilled water and treated by ultrasonic (400 W) for 60 min at room temperature. Then the mixture was heated to 50°C and maintained at this temperature for 10 min before a certain amount of alginate (1 wt.% with respect to the H_2_O) was slowly added to the above mixture. The suspension was kept stirring under vigorous speed (500 rpm) at 50°C for another 50 min in order to obtain a homogeneous mixture. To form the gel beads, the mixture was added dropwise into an aqueous CaCl_2_ (3 wt.%) solution at room temperature. The gel beads were left into CaCl_2_ aqueous overnight, and then the gel beads were separated and washed with distilled water to remove excess sodium and calcium ions. The gel beads were dried under vacuum overnight and calcined at 350°C under argon for 2 h with the ramp of 2°C/min.

**Figure 1:**
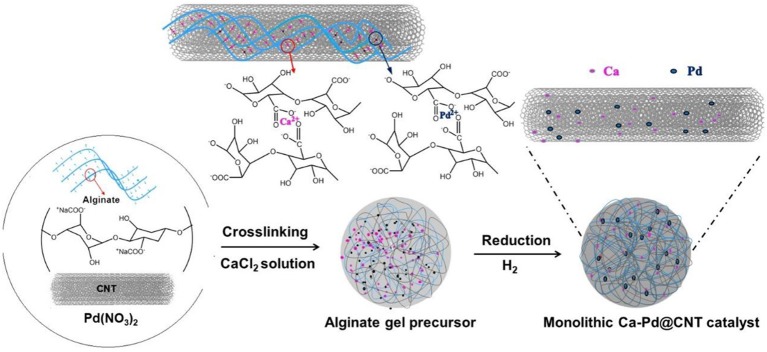
Schematic illustration of *in-situ* gel methodology for the synthesis of macroscopic shaped of Ca-Pd@CNT beads with Ca promoter.

Ca-Pd@CNT_HCl−x_ (x = 0, 2, 4) catalysts: Macroscopic shaped catalysts with 1 wt.% Pd loadings was synthesized by an *in-situ* process which was similar with aforementioned preparation method shown in Figure 1. Typically, the Pd(NO_3_)_2_·2H_2_O was added into the CNT-containing suspension accompanied with the alginate. The gel beads were dried under vacuum and then calcined at 350°C under argon for 2 h with the ramp of 2°C/min to obtain the Ca-Pd@CNT sample. The as-synthesized Ca-Pd@CNT sample was reduced under pure H_2_ at 400°C for 2 h. Furthermore, to investigate the influence of Ca promoter on the catalytic performance, the Ca-Pd@CNT sample was treated in dilute hydrochloric acid (1 mol/L) at room temperature for 2 and 4 h, respectively. HCl washed Ca-Pd@CNT sample were denoted as Ca-Pd@CNT_HCl−2h_ and Ca-Pd@CNT_HCl−4h_, respectively.

Pd/AC, Pd/CNT and macroscopic shaped Ca-Pd/CNT catalyst: A certain amount of Pd(NO_3_)_2_ solution was added into the active carbon (AC), powdered CNT and as-prepared CNT beads by wetness impregnation method. The samples were also dried under vacuum and calcined at 350°C under argon for 2 h with the ramp of 2°C/min, and then reduced under pure H_2_ at 400°C for 2 h before the CAL chemoselsctive hydrogenation reaction.

### Characterization

The morphology and compositions measurements were performed using a scanning electron microscopy (SEM) instrument (JSM-7800F, Japan), elemental mapping was operated on JSM-7800F with probe corrector at 15 kV. The textural properties of the samples were analyzed by N_2_ adsorption-desorption measurements using a Micromeritics ASAP 2020 plus sorptometer operated at −196°C. The samples were degassed under vacuum at 180°C for 6 h before the analysis in order to desorb moisture and adsorbed volatile species on the sample surface. The specific surface area was calculated according to Brunauer-Emmett-Teller (BET) method. Pore volume and average pore diameter were determined from using desorption branch by Barret-Joyner-Halenda (BJH) method. Inductively coupled plasma optical emission spectrometer (ICP-OES) was used to measure Pd and Ca element concentration by an IRIS Intrepid II XSP instrument. The particle size was obtained through using Transmission electron microscopy (TEM) technology which was performed on Titan Themis ETEM G3 (Thermofisher) with image corrector operating at 300 kV. High angle annular dark filed scanning TEM (HAADF-STEM) and elemental mapping were operated on Hitachi HF5000 with probe corrector at 200 kV. A small amount catalyst was crushed and ultrasonicated in ethanol, and then a drop was deposited onto a copper grid covered with a holey carbon membrane for analysis. X-ray photoelectron on spectroscopy (XPS) were recorded on a Thermofisher ESCALAB 250Xi spectrometer equipped with monochromatic Al KC (hυ = 1486.6 eV, 15 kV, 10.8 mA). The binding energies were calibrated by carbon deposit C1s with E_b_ = 284.6 eV.

### Catalytic Reaction

The liquid phase hydrogenation of cinnamaldehyde was performed in an autoclave reactor with pressure control system. The reaction process as follows: cinnamaldehyde was diluted in 1,4-dioxane to obtain a 0.45 mol/L reaction solution, and o-xylene was used as internal standard. Typically, 30 mg of catalyst was added into 5 mL reaction solution. The autoclave was then purged with hydrogen flow with four times to replace the air inside the autoclave and maintained the total pressure at 1 MPa at room temperature. Then, the autoclave was heated to 80°C in a water bath with a magnetic stirrer at a fixed rate (c.a. 800 rpm) to carry out the hydrogenation reaction. After the reaction, the reaction mixture was analyzed using gas chromatograph (GC-FID) equipped with an HP-5 column.

The HCAL selectivity was calculated according to the following equation:

(1) $$\begin{array}{l} {\text{HCAL}}_{\text{Selectivity}}\left(\%\right)=\\ \;\frac{\text{Moles of HCAL produced at reaction time}}{\text{Moles of CAL at converted at reaction time}}\times \;100\% \end{array}$$

## Results and Discussion

**Figure 2:**
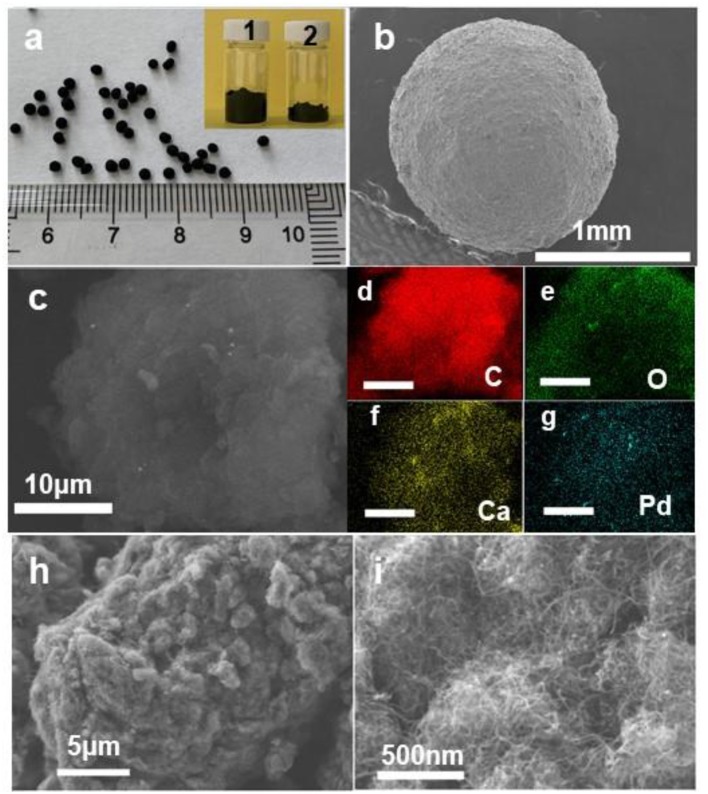
**(a)** Optical photos showing macroscopic shaped Ca-Pd@CNT catalyst. The inset image presents the volume of hybrid catalyst (1) and the compared initial CNT sample (2). **(b)** Low magnification SEM micrograph showing the gross morphology of the sphere structured catalyst. **(c–g)** SEM image and elemental mapping of Ca-Pd@CNT catalyst. **(h,i)** SEM images of Ca-Pd@CNT catalysts show the opened structure of as-synthesized beads.

The macroscopic shaped Ca-Pd@CNT catalyst was synthesized by an ionic induced crosslinking method. A series of Ca-Pd@CNT beads catalysts with controlled macroscopic shaping was prepared with around 0.9 wt.% Pd loading which was confirmed through ICP-OES analysis (Table 1). As shown in Figure 2, the uniform spherical beads with homogeneous diameter around 1.5 mm could be formed successfully through the *in-situ* gel process. Compared to the initial CNT powder, the apparent volume of the shaped catalyst is smaller due to the shrinkage during the drying process (the inset digital photo of Figure 2a). The energy-dispersive X-ray (EDX) elemental mapping images of Ca-Pd@CNT beads (Figures 2c–g) showed that the Pd and Ca are uniformly dispersed on the Ca-Pd@CNT beads, and only few of them are aggregated in the macroscale. The element composition Ca-Pd@CNT beads derived from EDS spectra is 0.45 at% (Ca) and 0.09 at% (Pd) in atomic percentage, which is a little lower compared to the actual mass loading from the ICP-OES results (vide infra). The medium and high-resolution SEM images clearly show that CNT in the composites entangled highly to form a large porosity dense network and the microscopic filamentous structure of CNT can be seen at high magnification. The above results indicated that highly dispersed particle could be obtained on the surface of CNT with a special macroscale structure through a simple preparation method.

**Table 1:** Specific surface area from N_2_ adsorption-desorption isotherms, exactly Pd, Na, and Ca mass loading and catalytic performance in selective hydrogenation of CAL^a^.

**Catalyst**	**SAA** **(m^**2**^/g)**	**Pd** **(wt.%)^b^**	**Ca** **(wt.%)^b^**	**Na** **(wt.%)^b^**	***d*_**TEM**_** **(nm)**	**CAL** **conversion** **(%)^b^**	**HCAL** **selectivity** **(%)**
Pd/CNT	170	0.74	0	–	5.5	40.3	75.8
Ca-Pd@CNT	209	0.92	4.33	0.0308	4.7	63.8	86.5
Ca-Pd@CNT_HCl−2h_	248	0.97	0.28	n.a.^c^	4.9	99.9	86.4
Ca-Pd@CNT_HCl−4h_	303	0.93	0.06	–	4.9	92.1	86.5

a *Reaction conditions: 10 bar H_2_, 80°C, 30 mg of catalyst, 5 ml 0.45 mol/L reaction mixture, dioxane as solvent, o-xylene as internal standard, reaction time: 30 min*.

b *The actual mass loading of Pd and Ca (wt.%) on the various Ca-Pd@CNT catalysts were determined by ICP-OES*.

c *Not available*.

**Figure 3:**
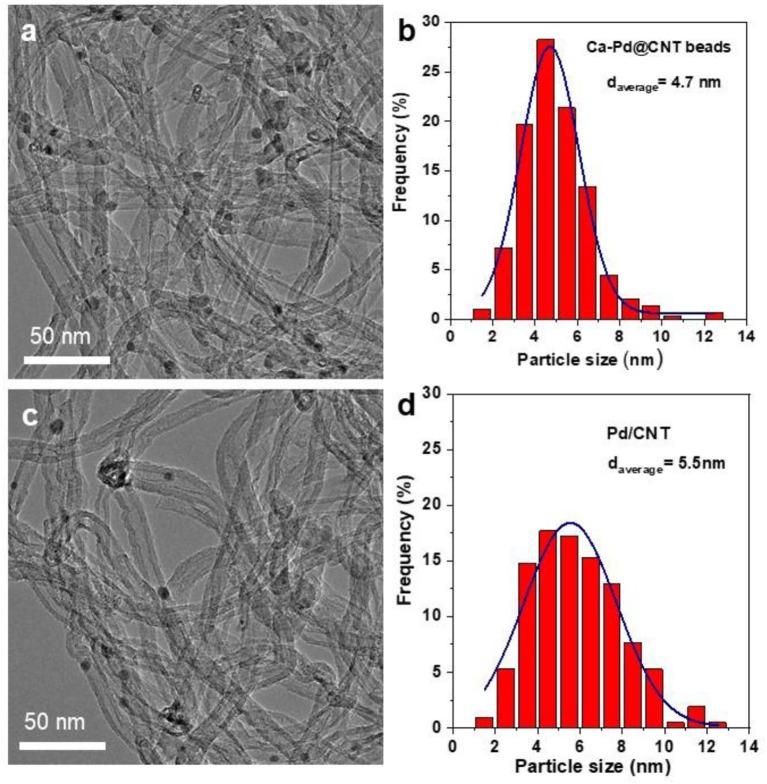
TEM images and Pd particle size distributions of **(a,b)** Ca-Pd@CNT beads and **(c,d)** Pd/CNT.

TEM image of as-synthesized Ca-Pd@CNT beads reveals that the Pd NPs anchored on the surface of CNT with relatively homogeneous size (Figure 3a). The particle size distribution of Ca-Pd@CNT beads is centered at around 4.7 nm, while the comparative sample, Pd NPs supported on powdered CNT (Pd/CNT), displays an average particle size of 5.5 nm with a broaden size distribution (Figure 3). Such relative homogeneous Pd particle size on the Ca-Pd@CNT beads could be directly attribute to the strong interaction between the metal NPs with Ca species as well as the graphitic layers of support surface.

**Figure 4:**
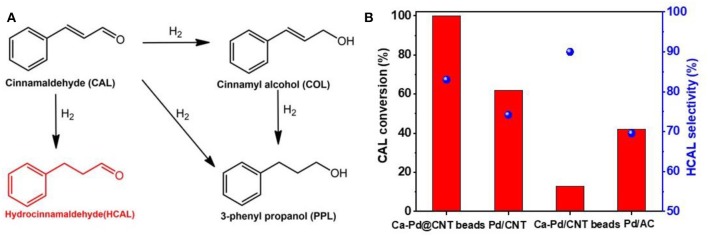
**(A)** Reaction scheme for the CAL hydrogenation. **(B)** CAL conversion and HCAL selectivity over Ca-Pd@CNT beads catalysts. Reaction conditions: 10 bar H_2_, 80°C, catalyst 30 mg, 5 ml 0.45 mol/L reaction mixture, dioxane as solvent, o-xylene as internal standard, reaction time: 120 min.

The chemoselective hydrogenation of cinnamaldehyde (CAL) to produce hydrocinnamaldehyde (HCAL) is selected as a probe reaction to investigate the structure-activity of the as-synthesized Ca-Pd@CNT beads catalysts (Figure 4A). For comparison, Pd/CNT beads, Pd/AC and Pd/CNT were also synthesized by impregnation method by using as-synthesized CNT beads, AC, and original CNT as support, respectively (see experimental section). Specially, Pd/AC sample presents the largest Pd average particle size at around 11.9 nm, which may be due to the weaken interaction between Pd NPs and active carbon (Jiang et al., 2016). The catalytic performances of CAL hydrogenation reaction and the corresponding HCAL selectivity over the various of supported Pd catalysts were displayed in Figure 4B. The selectivity of CAL to HCAL on Ca-Pd@CNT beads and Pd/CNT beads was relatively close (83.5 and 90.4%, respectively) under the same reaction conditions, whereas the CAL conversion on Ca-Pd@CNT beads (99.9%) was significantly higher than that of Pd/CNT beads (13.7%). It is obvious that the superior activity is obtained through the *in-situ* one-pot gel process. For Pd/AC and Pd/CNT catalysts, a lower HCAL selectivity was presented, 69.5 vs. 74.2%. Such lower selectively could be attributed to the bigger Pd particle size on Pd/AC catalyst (Xu et al., 2019). However, the possible reason for the relatively high HCAL selectivity compare to Pd/CNT catalyst could be that the presence of the additive Ca^2+^ ion during the catalyst preparation process (vide infra).

**Figure 5:**
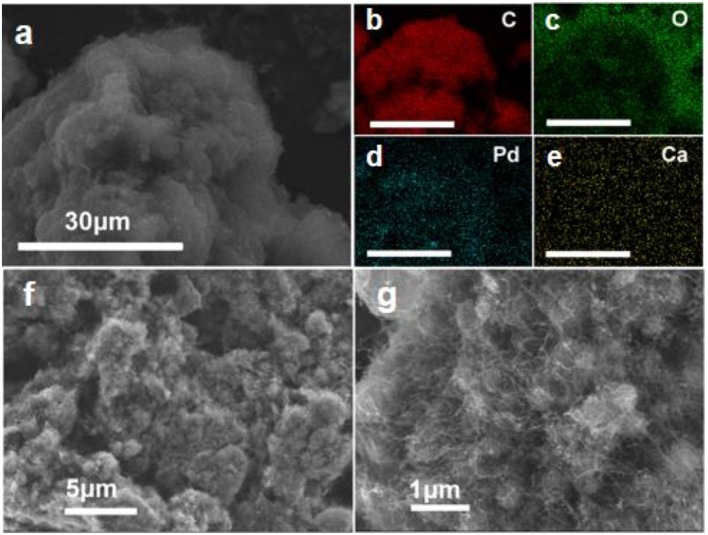
**(a–e)** EDS elemental mapping and **(f,g)** high resolution SEM images of Ca-Pd@CNT_HCl−2h_ catalysts.

In order to identify the detailed role of the Ca^2+^ ion in this chemoselective hydrogenation process, the acid etching experiment was conducted over Ca-Pd@ CNT catalyst. To leach the different amount of Ca^2+^ ion, the diluted hydrochloric acid (HCl) with 1.0 mol/L is used and the etching time is fixed at 2 and 4 h, respectively. As shown in Table 1, the Ca mass concentration is amount to 4.33, 0.28, and 0.06 wt.% for Ca-Pd@CNT, Ca-Pd@CNT_HCl−2h_, and Ca-Pd@CNT_HCl−4h_, respectively, from the ICP-OES analysis. It is worth noting that the Pd mass weight is maintained at the similar value, c.a. 0.9 wt.% (Table 1), after acid etching. It means that the residual of Ca could be removed by the diluted HCl solution. The high-resolution SEM images and EDS element mapping of Ca-Pd@CNT_HCl−2h_ beads (Figure 5) show the morphology of the sample have not been destroyed during the acid treatment and the relative low Ca concentration is appeared by EDS elemental mapping.

**Figure 6:**
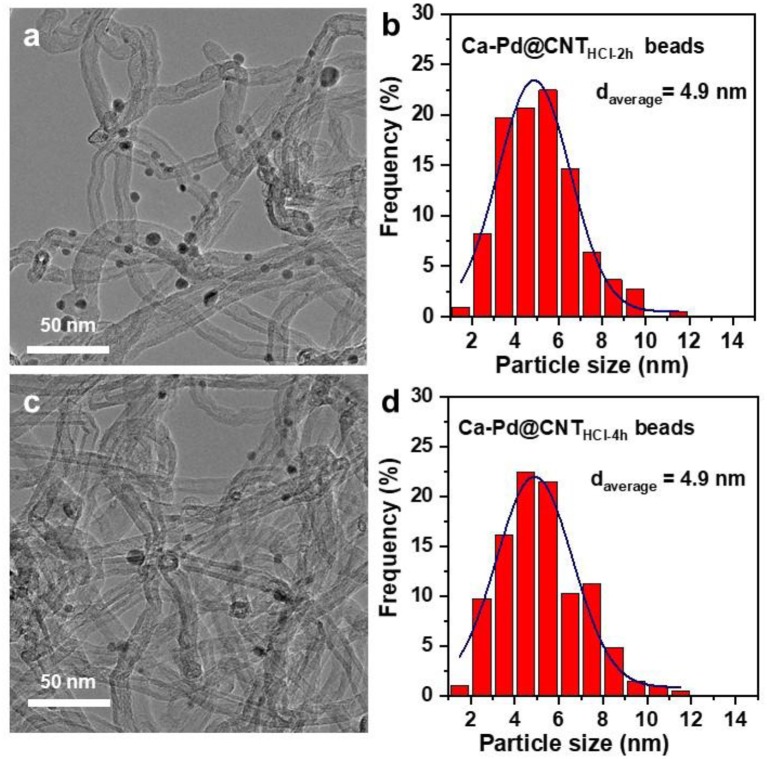
TEM images and Pd particle size distributions of **(a,b)** Ca-Pd@CNT_HCl−2h_, **(c,d)** Pd @CNT_HCl−4h_ catalysts.

**Figure 7:**
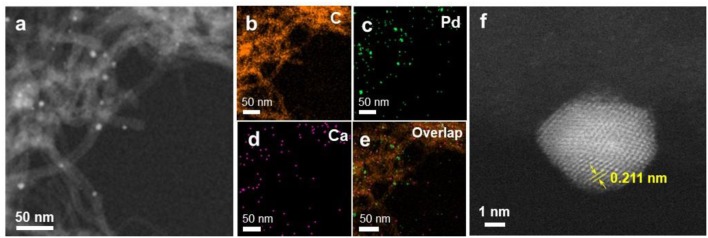
**(a–e)** HAADF-STEM image and EDS elemental mapping of C, Pd, and Ca on Ca-Pd@CNT_HCl−2h_ catalyst. **(f)** Atomic resolution HAADF-STEM image of typical Pd nanoparticles with defective structure on Ca-Pd@CNT_HCl−2h_ catalyst.

The particle size distribution of Ca-Pd@CNT beads with acid etching for 2 and 4 h was shown in Figure 6. As expected, the Ca-Pd@CNT_HCl−2h_ and Ca-Pd@CNT_HCl−4h_ had similar particle sizes distribution and particle shapes compared to that of untreated Ca-Pd@CNT beads (Figure 6), indicating that acid treatment has no significant influence on the Pd microstructure. The typical Ca-Pd@CNT_HCl−2h_ catalyst was further performed by atomic resolution STEM-HAADF for the microstructure analysis of calcium promoted Pd NPs (Figure 7). The EDS element mappings of Ca-Pd@CNT_HCl−2h_ catalyst reveal that the Ca species are anchored on the surface of CNT and interacted with Pd NPs. The lattice of Pd nanoparticle 0.211 nm, which is related to the (111) facet of Pd (Figure 7f). It is suggested that that the Ca does not enter the lattice of Pd nanoparticles.

The Ca-Pd@CNT_HCl−2h_ and Ca-Pd@CNT beads_HCl−4h_ catalyst are reduced at 200°C under H_2_ flow for CAL hydrogenation reaction. Compared to initial Ca-Pd@CNT catalyst, the CAL conversion of Ca-Pd@CNT_HCl−2h_ increases significantly from 63.8% reach to 99.9%, and the HCAL selectivity remains approximately (c.a. 86.5%). It can be clearly seen that a lower concentration of calcium ion could be beneficial for the enhancement of catalytic activity. When the content of Ca^2+^ further declined to 0.06 wt.%, the CAL conversion of Ca-Pd@CNT_HCl−4h_ is decreased to 92.1% with the similar HCAL selectivity 86.5%, which still presents superior catalytic activities compare to the Pd/CNT beads, Pd/CNT and Pd/AC catalysts. In addition, residual sodium could also plays the promotion role for noble metal nanoparticles in the hydrogenation reactions (King and Kelly, 2002; Kosydar et al., 2011; Long et al., 2019), where the sodium was also introduced during the synthesis process by using alginate as gel source. However, the Na concentration of the initial Ca-Pd@CNT remained extremely low, i.e., 0.0308 wt.%, and cannot be accurately detected by ICP-OES technique for the further acid treated sample (Ca-Pd@CNTHCl-2h). It could be revealed that Na could not affects the catalytic performance in this catalyst system. Thus, the results clearly demonstrate that in presence of Ca promoter can significantly increase the CAL hydrogenation activity, and the various Ca concentration could induce the different CAL hydrogenation ability. The extra Ca species covered on the surface of Pd NP could be etched during the moderate acid treatment process that result highly Pd active site exploration.

**Figure 8:**
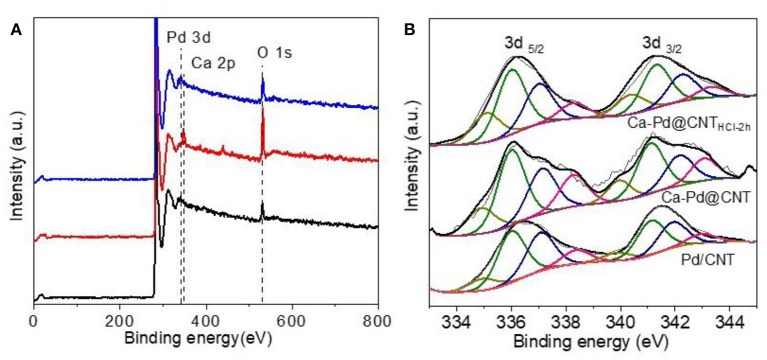
**(A)** XPS survey spectra and high-resolution Pd 3d spectra of reduced Pd/CNT powder, Ca-Pd@CNT beads, and Ca-Pd@CNT_HCl−2h_ beads. **(B)** The deconvoluted Pd 3d spectrum of reduced Pd/CNT powder, Ca-Pd@CNT beads, and Ca-Pd@CNTHCl-2h beads.

XPS survey spectra of Pd-decorated CNT are shown in Figure 8A. For the Pd/CNT sample, the XPS spectral show that distinct C 1s, O 1s, and Pd 3d peaks, and no other elements peaks are detected. The strong Pd 3d peaks are detected over the sample, suggesting that the formation of numerous Pd nanoparticles loaded on CNT carrier. In addition to the strong C 1s, O 1s, and Pd 3d peaks of Ca-Pd@CNT and Ca-Pd@CNT_HCl−2h_ beads, which also has a strong Ca 2p and Ca 2s peak. The XPS analyses were conducted to reveal the electronic structure of the Pd species with or without the interaction of Ca ion (Rao et al., 2017). The XPS survey spectra of all samples show the expected elements (i.e., C, O. Pd) as well as the Ca that are derived from the cross-linking process for spherical Ca-Pd@CNT beads and Ca-Pd@CNT_HCl−2h_ catalysts (Figure 8A). The deconvoluted Pd_3d_ XPS spectra of Pd supported catalyst (Figure 8B) present two main peaks at about 335.35 and 340.35 eV, corresponding to the doublet of Pd 3d_5/2_ and 3d_3/2_, respectively (Rao et al., 2017). The deconvolution of core level Pd3d_5/2_ revealed the presence of predominant metallic phase (Pd^0^, 335.1 eV), oxide state metal (Pd^2+^, 337.0 eV) and a satellite peak (338.3 eV) (Pillo et al., 1997; Zhao et al., 2015; Arrigo et al., 2016). The oxide state Pd^2+^ was ascribed to the oxidation occurs on the surface of Pd NPs during the passivation process by diluted oxygen gas mixture (0.5 vol.% O_2_ in helium). Furthermore, there was an apparent peak between Pd^0^ and Pd^2+^ attribute to the electron transfer from Pd to the support, and then to produce the electron-depleted palladium species (Pd^δ+^) (Cárdenas-Lizana et al., 2013; Arrigo et al., 2016). As shown in Table 2, the lowest molar ratio of Pd^0^/Pd^δ+^ is presented in Ca-Pd@CNT_HCl−2h_ sample indicating that a certain amount of Ca promoter is in favor of forming Pd^δ+^ species which may in presence of electron transfer from Pd to Ca (Song et al., 2006), and thus accounts for the superior hydrogenation activity.

**Table 2:** Elemental analysis of Pd *3d*_5/2_ XPS spectra on monolithic Pd/CNT, Ca-Pd@CNT, and Ca-Pd@CNT_HCl−2h_ catalysts.

**Sample**		**Pd 3d**_****5/2****_			
	**Pd**^****0****^		**Pd**^****σ+****^		**Pd^**0**^/Pd^**δ+**^**
	**BE/eV**	**at%**	**BE/eV**	**at%**	
Pd/CNT	335.1	9.9	336.0	22.0	0.45
Ca-Pd@CNT	334.9	8.7	336.0	25.0	0.35
Ca-Pd@CNT_HCl−2h_	334.9	6.2	336.0	26.7	0.23

## Conclusion

In conclusion, the *in-situ* gel process is employed successfully to prepare a controllable macroscopic shaped nanocarbon material containing Pd nanoparticle for chemoselective hydrogenation of CAL. The Ca promoter in the Ca-Pd@CNT beads is introduced by the interaction between Ca^2+^ ions and the hydroxyl and carboxyl groups of alginates during the cross-linking of alginate chains. As a result, the CAL hydrogenation activity is significantly enhanced in presence of Ca species through modifying the electron structure of Pd species. Compared to other prepared carbon-supported catalysts, the Ca-Pd@CNT_HCl−2h_ with the moderate Ca loading displays the superior CAL hydrogenation activity as well as the HCAL selectivity which could be attributed to the lower Pd^0^/Pd^δ+^ ratio in the catalyst. Besides, the controllable monolithic structure could endow the carbon-based catalyst easy handling during the reaction and recovery process in the liquid-phase reaction. Moreover, such macroscopic process with *in-situ* introduced active metal species may also be applied in gas-phase reaction systems which could reduce the overall pressure drop across the packed bed comparing with the powered carbon-based catalysts.

## Data Availability Statement

The raw data supporting the conclusions of this manuscript will be made available by the authors, without undue reservation, to any qualified researcher.

## Author Contributions

YM and LF prepared the catalysts, conducted the characterization, reaction tests, and analyzed the data. JD prepared a part of catalysts. ZG conducted the HR-TEM analyses. CP-H contributed to thorough discussions on this work. YL designed the experiments and wrote the paper.

### Conflict of Interest

The authors declare that the research was conducted in the absence of any commercial or financial relationships that could be construed as a potential conflict of interest. The handling editor declared a past co-authorship with one of the authors (YL).
